# A new developmental mechanism for the separation of the mammalian middle ear ossicles from the jaw

**DOI:** 10.1098/rspb.2016.2416

**Published:** 2017-02-08

**Authors:** Daniel J. Urban, Neal Anthwal, Zhe-Xi Luo, Jennifer A. Maier, Alexa Sadier, Abigail S. Tucker, Karen E. Sears

**Affiliations:** 1 School of Integrative Biology, University of Illinois, 505 S Goodwin Avenue, Urbana, IL 61801, USA; 2 Carl Woese Institute for Genomic Biology, University of Illinois, 1206 W Gregory Drive, Urbana, IL 61801, USA; 3 Department of Craniofacial Development and Stem Cell Biology, King's College London, London, UK; 4 Department of Organismal Biology and Anatomy, University of Chicago, Chicago, IL 60637, USA

**Keywords:** apoptosis, marsupial, Meckel's cartilage, origin of mammals, TGFB signalling

## Abstract

Multiple mammalian lineages independently evolved a definitive mammalian middle ear (DMME) through breakdown of Meckel's cartilage (MC). However, the cellular and molecular drivers of this evolutionary transition remain unknown for most mammal groups. Here, we identify such drivers in the living marsupial opossum *Monodelphis domestica*, whose MC transformation during development anatomically mirrors the evolutionary transformation observed in fossils. Specifically, we link increases in cellular apoptosis and *TGF-BR2* signalling to MC breakdown in opossums. We demonstrate that a simple change in *TGF-β* signalling is sufficient to inhibit MC breakdown during opossum development, indicating that changes in *TGF-β* signalling might be key during mammalian evolution. Furthermore, the apoptosis that we observe during opossum MC breakdown does not seemingly occur in mouse, consistent with homoplastic DMME evolution in the marsupial and placental lineages.

## 1. Introduction

The evolutionary origin of the definitive mammalian middle ear (DMME) is often cited as a textbook example of evolutionary transformation [1–3]. Reptiles and pre-mammalian synapsids possess multiple bones in the jaw but only a single bone (stapes) in the middle ear. By comparison, all mammals possess a single dentary bone in the jaw, and multiple bones in the middle ear, namely the malleus, incus and ectotympanic, in addition to the stapes. The incorporation of multiple bony elements into the middle ear increased the hearing sensitivity of mammals, most notably to high-frequency sounds, a trait that is thought to have benefited the mammalian lineage [4,5]. In addition, the DMME is one of the few, key bony hallmarks for the origins of Mammalia in the fossil record [2,6,7].

Significant developmental and palaeontological evidence suggests that the malleus and incus of the mammalian ear evolved from the articular and quadrate of the reptilian jaw, respectively. Beautiful fossils encompassing the reptile to mammal transition document the transformation of the reptilian jaw elements into mammalian middle ear ossicles (for an overview, see [3]). A similar transformation occurs during mammalian development, and has been documented in the embryos of multiple mammal species [1–3,8–10]. Furthermore, developmental genetic evidence from mouse [9,11] and chick [12] has confirmed that the articular and quadrate (malleus and incus, respectively) initially form as a single cartilaginous condensation that expresses *Bapx1* and is physically linked to Meckel's cartilage (MC) of the jaw. However, while the fact that jaw elements transform into middle ear ossicles over developmental and evolutionary time is generally accepted, the specific developmental processes driving this transformation remain largely unknown. This gap in knowledge significantly impairs our understanding of processes that have fundamentally shaped the course of mammalian evolution.

In this study, we investigate the cellular and molecular processes driving a crucial first step in the evolution of the mammalian middle ear ossicles (i.e. malleus, incus)—their disconnection from the jaw. This occurred ancestrally in at least three lineages of Mesozoic mammals (monotremes, multituberculates and therians (marsupials + placentals)) [13,14], and occurs early in the development of extant mammals, through the breakdown of the part of MC connecting the malleus and ectotympanic to the dentary [15–18]. Although it was once suggested that the physical process of brain expansion separated mammalian middle ear elements from the dentary [19], this claim has since been refuted [16,20–22]. To explore the disconnection of the middle ear elements from the jaw, we thus investigated the processes driving MC breakdown by taking advantage of the unique development of a living marsupial mammal, the grey, short-tailed opossum *Monodelphis domestica*.

Like other marsupials, opossums are born in a premature state [23]. Of most relevance to DMME development, the malleus and incus of newborn opossums remain attached to the jaw in a reptile-like morphology. The malleus remains physically connected to the jaw for a few weeks after birth. At that point, MC breaks down, and the malleus and incus detach from the jaw. The squamosal-dentary joint then becomes dominant and the malleus and incus become part of the DMME [15,17,18]. The malleus and incus are also initially attached to the jaw and detach through MC breakdown in placentals, such as mice. However, the mouse malleus and incus achieve their adult positions by birth, and MC breakdown occurs shortly thereafter (between P1 and P2) [8]. The malleus and incus are therefore never a part of the postnatal mouse jaw. Furthermore, while the gestational times are not equal (mice approx. 20 days, opossums approx. 14 days), the separation of MC from the jaw is still distinctly delayed in opossums, equivalent to occurring after two weeks of postnatal age in mice. As the development of the opossum DMME anatomically mirrors the progression of DMME evolution in the mammalian fossil record, the opossum provides an exceptional, living model system for MC breakdown.

In this report, we use anatomical, cellular, gene expression and functional assays to provide the first evidence for the cellular and molecular basis for MC breakdown in opossum. Specifically, we produce evidence correlating increases in apoptotic cell death and functionally linking changes in *TGF-β* signalling to the breakdown of MC, and the associated freeing of the middle ear ossicles from the jaw, in opossums. Given the similarity between opossum development and mammalian evolution, it is possible that similar processes contributed to at least some instances of MC breakdown and the associated formation of the DMME during the reptile to mammal transition.

## 2. Material and methods

### (a) Sample collection

Opossum (*M. domestica*) pups were collected from a breeding colony maintained by the Sears Lab at the University of Illinois at Urbana-Champaign (UIUC) [24], in accordance with fully approved IACUC procedures. Specimens were euthanized via carbon dioxide inhalation, followed by cervical dislocation, and heads were severed at approximately the third cervical vertebrae.

### (b) Micro-CT

Opossum pups were first collected on postnatal day 1 and at 5-day intervals thereafter (day 5, 10, 15…), up to postnatal day 35 (*N* = minimum of three per stage), and heads were skinned and fixed in 4% paraformaldehyde overnight at 4°C. On the following day, specimens were dehydrated in a methanol series into 100% MeOH, and stored at −20°C. X-ray absorption in cartilage is similar to that of soft tissue and can be difficult to differentiate. To account for this, we dried the heads using a Tousimis 931 Series Critical Point Dryer with a critical point set more than 1072 psi and more than 31°C for 15–20 min depending upon head size to increase the visualization of cartilage tissues. Critical point drying allows the tissue to dry rapidly at high pressure and thereby preserves size and shape. Imaging of the heads was conducted on an Xradia Bio MicroCT (MicroXCT-400). X-ray specifications were set to source voltage 20–40 kV, power 4.0–8.0 W and current 200 µA, depending upon the extent of ossification for each specimen. We used a 0.5× lens with 1× binning, and captured approximately 800–900 images per sample. The developing cartilage elements of the middle ear and jaw were hand-traced for every individual MicroCT image to insure correct placement of all relevant cartilaginous structures (as cartilage structures are often difficult to visualize using auto-reconstructions), and the tracings incorporated into overall skull reconstructions. Three-dimensional reconstruction and analysis was conducted using Amira 5.6.0 software (FEI Visualization Sciences Group, Bordeaux, France).

### (c) Immunofluorescence

In total, 16, 18 and 20-day old opossums were cryosectioned, and put through immunofluorescence (IF) staining to highlight apoptosis (using EMD Millipore ApopTag Fluorescein In Situ Apoptosis Detection Kit (S7110), an indirect TUNEL method), autophagy (using Anit-LC3B antibody (ab51520 from Abcam), and cellular proliferation (using Phosphohistone H3 (Ser10) antibody from Cell Signaling Technology) [25,26]. More detailed IF methods can be found in the electronic supplementary Materials and Methods.

### (d) Gene expression assays

Additional samples were cryosectioned to collect tissue for RNA-Seq. *N* = 3 specimens were collected for each stage (16, 18 and 20 day) and an Arcturus Veritas Microdissection Instrument was used to laser capture microdissect (LCM) the MC and malleus (with minimal surrounding perichondrium) focusing on their connection area. RNA isolation was completed using an Arcturus PicoPure RNA Isolation Kit, and the Clontech SMARTer Ultra Low Input RNA Kit for RNA amplification. RNA-Seq Libraries were constructed using a Nextera XT DNA Sample Preparation Kit. High-throughput sequencing was conducted on an Illumina HiSeq 2500, at the W.M. Keck Center for Comparative and Functional Genomics at the University of Illinois [27]. RNA-Seq analysis was conducted on the UIUC Web-based Galaxy [28] platform (galaxy.illinois.edu), using the Tuxedo protocol [29,30]. The Database for Annotation, Visualization and Integrative Discovery (DAVID) Bioinformatics Resource (david.ncifcrf.gov) [31] was also used to identify Gene Ontology (GO) terms. Resulting datasets will be deposited and made freely available in NCBI upon acceptance and publication. For more details about RNA-Seq methods, see electronic supplementary Materials and Methods.

The differential expression of select RNA-Seq genes, namely *TGFbr2* and *WISP1*, was confirmed using fluorescence *in situ* hybridization (FISH, probes from Molecular Instruments, XM_007505215 accession number for TGFBR2, XM_007488362.2 accession number for WISP1) [32] on cryosectioned slides from 16, 18 and 20 day specimens. For details, see the electronic supplementary Materials and Methods.

### (e) TGF-β knockdown

We performed intraperitoneal injections of TGF-β neutralizing-antibody (TNA; TGFb1,2,3—MAB1835 from R&D Systems) or control solution (TGF-β vehicle) into opossum pups every day from P16 to P22 [33]. The antibody was administered at 10 ng kg^−1^. Pups were euthanized on postnatal day 22. To confirm the knockdown of *TGF-β* signalling, we cryosectioned the middle ear regions of TNA and control pups at P22, and performed IF for anti-p-Smad2 (Cell Signaling Technology) [34]. pSMAD is a downstream protein of *TGF-β* signalling [34–36]. MC morphology was visualized using micro-CT scanning and clearing and staining [34], and apoptotic cells (Cell Signaling Technology) using TUNEL on cryosectioned middle ear sections. The length and width of the skull and ectotympanic was measured in triplicate and averaged for each micro-CT scanned TNA and control pup, and statistically compared using the Wilcoxon–Mann–Whitney rank sum test [26]. For details, see the electronic supplementary Materials and Methods.

## 3. Results

To provide a reference time point for further assays, we first sought to confirm the timing of the separation of MC and the malleus during opossum development. Using refined micro-CT scanning, we found that the first separation between MC and the malleus appears at P20 (electronic supplementary material, figure S1).

**Figure 1.**
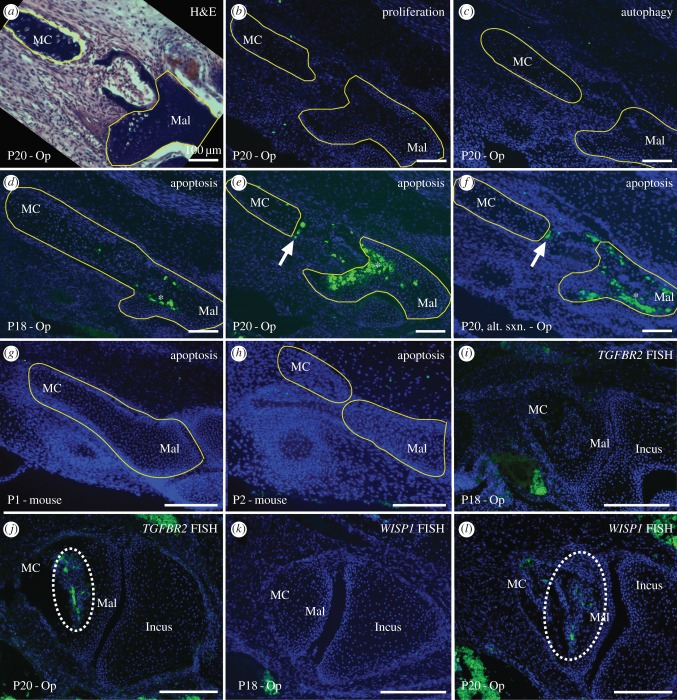
Middle ear sections of opossum postnatal day (P) 18 (*d*,*i*,*k*) and P20 (*a*–*c*,*e*,*f*,*j*,*l*), and mouse P1 (*g*) and P2 (*h*) in dorsal view, showing the MC and malleus (Mal) separation. All except (*a*) are counterstained with DAPI (nuclear stain, blue cells). (*a*) Haematoxylin and eosin stain for anatomical reference, showing MC on the left and Mal on the right. (*b*) Proliferation IF (green cells) shows few proliferating cells, and none near MC breakdown. (*c*) Similarly, autophagy IF (green cells) shows no autophagy near MC breakdown. (*d*–*f*) Apoptosis TUNEL (green cells) shows significant death in the anterior malleus (asterisks in *d*–*f*), that is associated with ossification rather than MC breakdown. However, a distinct apoptotic line is present along the MC's posterior edge at P20 (white arrows in *e*,*f*). (*g*,*h*) Apoptosis TUNEL (green cells) in mice shows no apoptosis associated with MC breakdown. (*i*–*l*) FISH for *TGFBR2* (*i*,*j*) and *WISP1* (*k*,*l*) (green cells, circled with broken white lines) shows gene expression during MC breakdown at P20 (*j*,*l*), but not before (*i*,*k*). Scale bars, 100 µm.

We next performed IF on middle ear sections to assess the cellular processes of apoptosis, autophagy and proliferation in the opossum MC during its breakdown at P20. We observed no change in the levels of cellular proliferation or autophagy in tandem with MC separation at P20 in opossum (figure 1; electronic supplementary material, figure S3). By contrast, TUNEL staining reveals a clear line of apoptotic cells along MC's posterior edge at this time (figure 1). The line of cell death traverses cell layers, always adheres to the posterior edge of the degrading MC, and is not present before P20 (figure 1). The line of cell death is also definitively distinct from ossification-related apoptosis occurring in the malleus, and the portion of MC adjacent to the line of cell death does not ossify in extant mammals [37]. To facilitate comparison of our cell death results from opossum with those from mouse, we also performed TUNEL on middle ear sections of P1 and P2 mice (the time of MC breakdown in mouse [8]). Unlike the situation in opossum, we did not observe any apoptotic cells in the mouse MC during its breakdown (figure 1, note that apoptosis is not observed in the mouse malleus, as in opossum, as MC breakdown in mouse occurs before the onset of malleus ossification).

To investigate the molecular drivers of MC breakdown in opossums, we used LCM to excise MC and its perichondrium immediately anterior to the malleus from P16 and P18 (before breakdown), and P20 (breakdown initiation), and performed RNA-seq on the collected tissues (electronic supplementary material, figure S2). We identified fewer than 300 genes (less than 3%; electronic supplementary material, table S1) that are differentially expressed by a log-fold change of greater than or equal to 2 at and before P20. Our GO term analysis identified significantly upregulated genes with roles in processes including apoptosis, including *TGFBR2* [38] and *WISP1* [39,40], as well as roles in bone breakdown and cartilage resorption, such as *MMP9* [41,42], *ACP5* [43,44] and *CTSK* [45]. Given our cellular results, we chose to further investigate the identified genes with roles in processes including apoptosis.

We performed FISH for *TGFBR2* and *WISP1* on opossum middle ear sections from P16, P18 and P20. We found that both genes are either not expressed or are expressed at very low levels in the MC region at P16. However, both genes are strongly expressed in the MC region at P20 (figure 1). The upregulation of these genes during MC breakdown is consistent with them having a role in this process.

**Figure 2.**
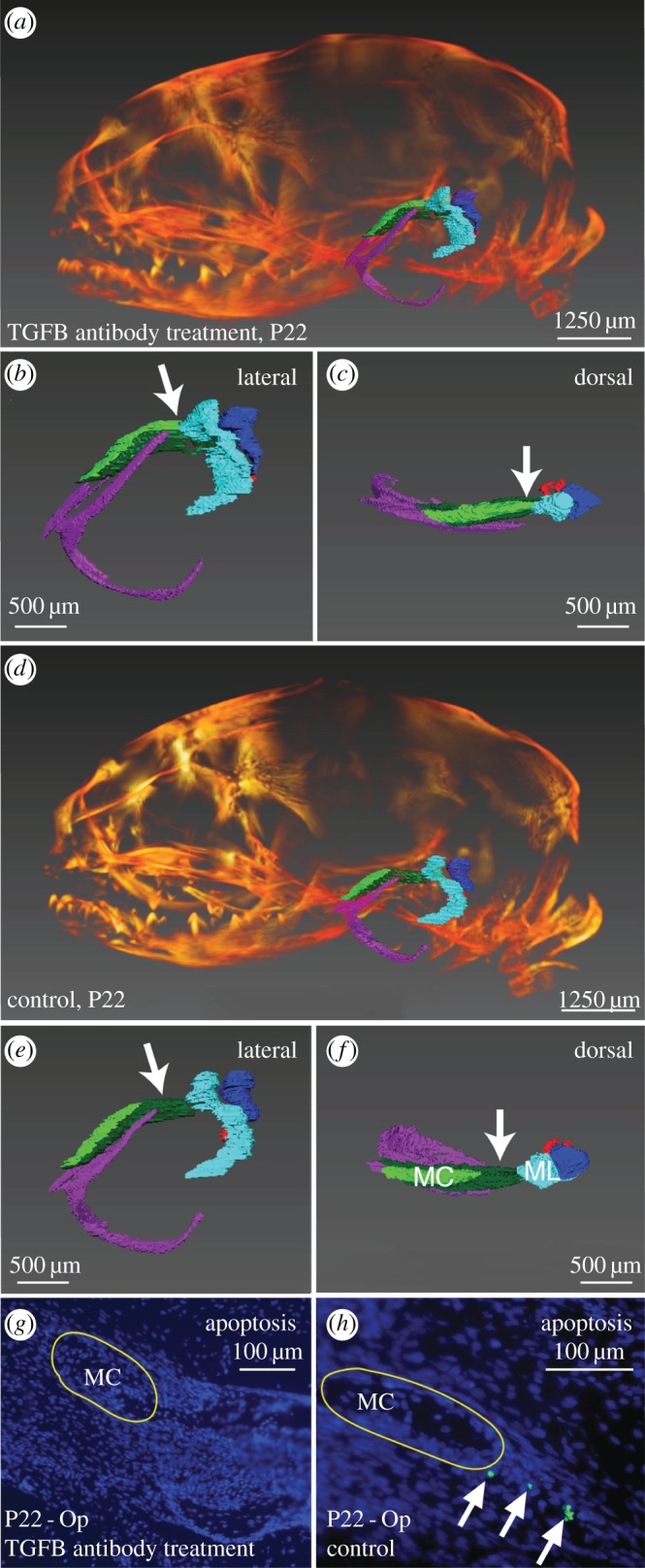
Micro-CT reconstructions (*a*–*f*) and dorsal sections (*g*,*h*) of *TGF-β* neutralizing-antibody (TNA) treated (*a*–*c*,*g*) and control (*d*–*f*,*h*) opossum pups at postnatal day (P) 22. Middle ear elements are coloured for micro-CT (MC, light green; ectotympanic, purple; goniale, dark green; malleus, light blue; incus, dark blue; stapes, red). (*A*–*f*) Micro-CT reconstructions of the whole skull in TNA (*a*) and control pups (*d*), and of isolated middle ears in lateral (TNA = *b*, control = *e*) and dorsal (TNA = *c*, control = *f*) views. In TNA pups, MC (light green) is contiguous with the malleus (light blue), white arrows (*b*,*c*) show connection. By contrast, the MC and malleus have disconnected in controls, white arrows (*e*,*f*) show separation. (*g*,*h*) Apoptosis TUNEL (green cells) on MC sections shows MC-related apoptosis is absent from TNA pups (*g*), but present (white arrows) in controls (*h*). Mal = malleus.

To further test the hypothesis that *TGF-β* signalling contributes to MC breakdown in opossum, we injected a TNA or control solution (TGF-β vehicle) into opossum pups and assessed the impact on phenotype [33]. Consistent with *TGF-β* signalling inhibition, significantly more pSMAD-positive cells are present in control than TNA treated pups (*p* = 0.0001, electronic supplementary material, figure S3). Control pups also display a line of TUNEL-positive apoptotic cell death at the leading edge of MC breakdown, while apoptotic cells are absent from TNA pups (figure 2). In addition, micro-CT (figure 2) and clearing and staining (electronic supplementary material, figure S4) results show that MC and the malleus of TNA remain connected at P22, while those of controls have separated. All other DMME components have similar structures in control and TNA pups (figure 2; electronic supplementary material, figure S4). For example, the lengths and heights of the ectotympanic and skull overlap for TNA and control pups, and do not significantly differ between the two (skull length, *p*-value = 0.15; skull height, *p*-value = 0.57; ectotympanic length, *p*-value = 0.15; ectotympanic height, *p*-value = 0.15).

## 4. Discussion

Our results suggest that MC breakdown begins at P20 in opossums, consistent with prior studies which found that: MC and malleus are separated by the end of the third week of postnatal development [19], and that by P20 the middle ear ossicles are no longer connected to the dentary [46]. Our IF studies of developing opossums at this stage are consistent with an increase in apoptotic cell death, but not changes in cellular autophagy or proliferation, contributing to the initiation of MC breakdown and the separation of the middle ear ossicles and jaw during opossum development. In contrast to these findings, past studies have suggested a role in MC breakdown for cellular autophagy but not apoptosis in mice [11,47,48], and one concurrent study a role for clast-activity in mice and opossums in MC breakdown [49]. Our own TUNEL assays on mice middle ear regions confirm the absence of apoptotic cells during MC breakdown in this species, and our RNA-Seq results are consistent with an increase in the expression of clast-activity genes during opossum MC breakdown. Taken together, these findings suggest that while some cellular processes associated with MC breakdown may be conserved in mice and opossums (e.g. clast activity), others are likely distinct (e.g. apoptosis).

**Figure 3.**
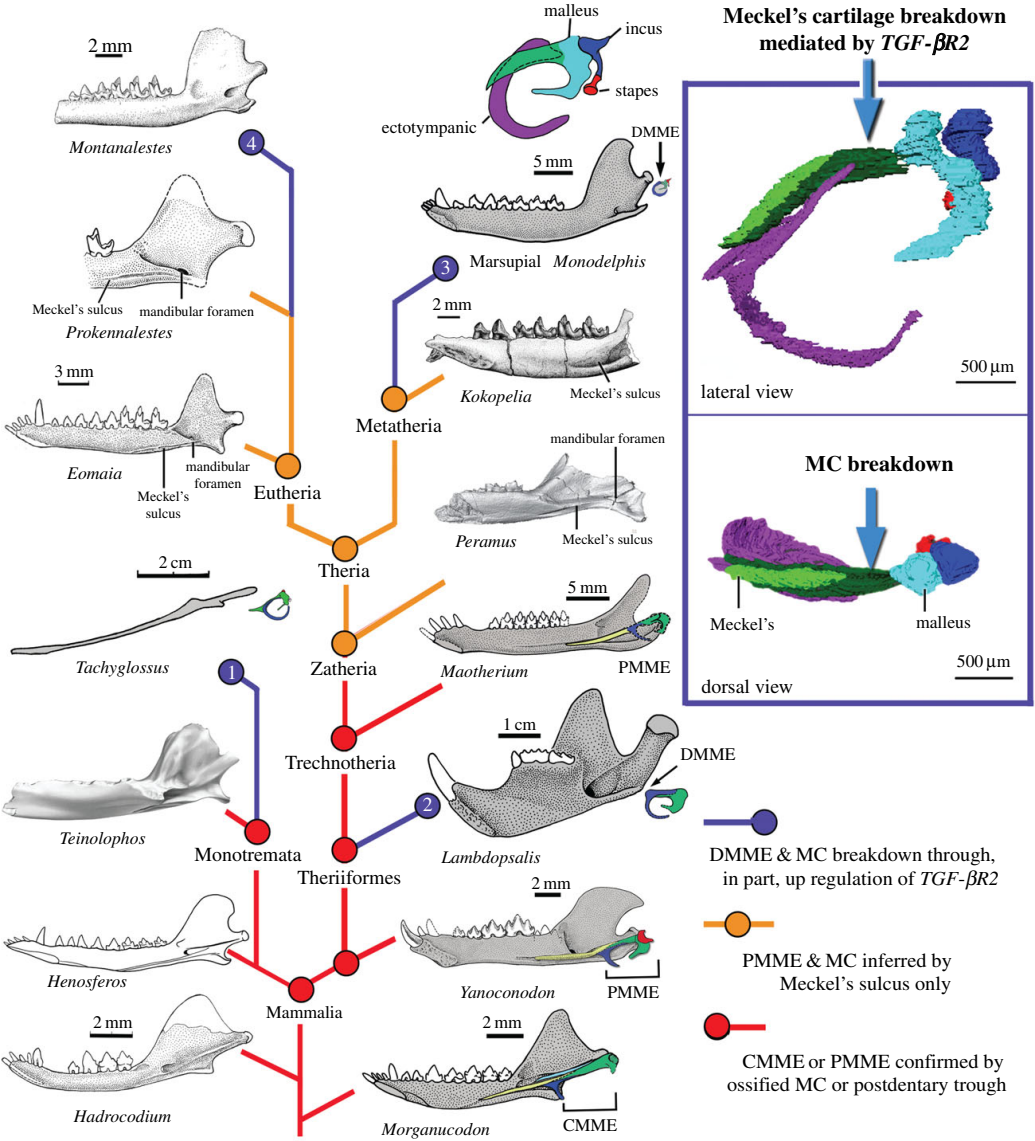
We hypothesize that DMME detachment from the jaw, via MC breakdown mediated, in part, by *TGF-βR2* upregulation (right panel), occurred independently in four lineages: extant Monotremata (Node 1), multituberculates (Node 2), and likely also in extant marsupials (Node 3) and extant placentals (Node 4) (blue nodes and lines). In ancestral mammaliaforms, the intact Meckel's element of the cynodont mandibular middle ear or of the partially mandibular middle ear (in *Yanoconodon* and *Maotherium*) is attached to Meckel's sulcus (red nodes and lines). Meckel's sulcus is therefore an indicator of MC-mediated connection between the jaw and middle ear. Meckel's sulcus is a prominent feature of zatherians (*Peramus*), and crown therians through some stem metatherians (*Kokopelia*) and eutherians (*Eomaia, Prokennalestes*). Thus, it may be inferred that zatherians and therians ancestrally had an MC-mediated connection between the jaw and middle ear (brown nodes and lines). Assuming that basal eutherians and metatherians had PMMEs, the DMME must have arisen separately in extant placentals (through increased clast activity and autophagy) and in extant marsupials (through increased clast activity and apoptosis). Phylogeny from [3,53,54].

The differences between clast-mediated MC breakdown in mice, and apoptosis- and clast-mediated breakdown in opossums, are consistent with developmental systems drift [50] and/or independent DMME acquisition in placental and marsupial lineages. Both hypotheses are intriguing, and the latter is supported by fossil evidence from the Meckel's sulcus. Meckel's sulci are structurally associated with an MC–middle ear connection in many Mesozoic mammals [3,22,51]. Two stem eutherians, *Prokennalestes* [51] and *Eomaia* [52], which are related to modern placentals, have clearly preserved Meckel's sulci (figure 3) [55]. Similarly, the stem metatherian *Kokopellia* also has a distinctive Meckel's sulcus (figure 3) [56]. Enlarged Meckel's sulci are also present in the immediate outgroups of the metatherian–eutherian clade, such as the cladotherians *Peramus* [57] and *Palaexonodon* [53]. Independent studies of these stem eutherians, metatherians and their outgroups have all determined that the Meckel's sulci in these forms are for the MC, not other soft-tissue structures [51–53,55–57], which is corroborated by our own survey of the mandibles of other extant therians [49]. Thus, it can be inferred that the MC and ear were connected in basal eutherians and metatherians, and that MC breakdown occurred separately in the eutherian-placental and metatherian-marsupial lineages through at least partially distinct cellular patterns (figure 3, for an alternative scenario see the electronic supplementary material, figure S5).

This study also identified several genes that are significantly upregulated during MC breakdown in opossums, including *TGFBR2*. This is of note, as *TGF-β* signalling, through its receptor *TGFBR2*, has been shown to significantly impact MC and middle ear development in mouse [58,59]. In mouse, *TGF-β* signalling contributes to MC patterning, and null mutations in the pathway can cause premature MC ossification such that the jaw and middle ear ossicles remain connected [34,58,59]. In fact, the phenotype of the ossified MC in *Tgfbr2^fl^*^/*fl*^*;Wnt1-Cre* mutant mice is similar to the ossified MC in some early mammals [57]. This led to the hypothesis [3,60] that changes in *TGF-β* signalling had been involved in the repeated evolution of the DMME from the ancestral jaw structure of pre-mammalian synapsids and reptiles.

To test the hypothesis that *TGF-β* signalling contributes to MC breakdown in developing opossums, we experimentally reduced *TGF-β* signalling levels and observed the impact on the opossum phenotype. The phenotype of the resulting opossums is particularly striking and is reminiscent of that of ancestral mammals. By reducing *TGF-β* signalling by protein knockdown, we eliminated the cellular apoptosis that normally marks the leading edge of MC breakdown in developing opossums, and the MC breakdown that normally separates the MC and malleus. All other DMME components have similar structures in control pups and pups in which *TGF-β* signalling has been experimentally reduced, suggesting that the reduction of *TGF-β* signalling does not significantly impact the overall rate or pattern of development. Taken together, these results suggest that *TGF-β* signalling in opossums is sufficient to drive MC apoptosis and breakdown, and the disconnection of the middle ear ossicles and jaw, with almost no obvious pleiotropic phenotypic effects on surrounding middle ear structures.

Given the striking anatomical similarities between opossum middle ear development and mammalian middle ear evolution [3,19], it is possible that similar changes in *TGF-β* signalling and apoptosis contributed to DMME evolution in at least some mammalian lineages. Furthermore, our results demonstrate that even a small change in a single signalling pathway (e.g. *TGF-β*) can trigger MC retention, and can do so without disrupting the development of other jaw and ear structures (electronic supplementary material, figure S4). The apparent ease of this transformation provides a possible explanation for the frequent gains and losses of an ossified MC in mammalian lineages proposed from study of the fossil record (figure 3; electronic supplementary material, figure S5). A recent study of tympanic membrane (TM) development shows that the TM attached to the ectotympanic and the malleus is transformed in embryogenesis by shifting of the TM precursor to the lower jaw components in mammals, in contrast to non-mammalian extant diapsids in which the TM precursor shifts its attachment to upper jaw components in embryogenesis [61]. Thus, TM development is convergent in these amniote clades. New evidence that the disconnection of the ectotympanic and malleus from the MC occurred perhaps as many as four times in mammalian evolution (figure 3), or at least three times (electronic supplementary material, figure S5), is consistent with the wider evolutionary homoplasies of the middle ears of extant amniotes.

## Supplementary Material

Supplementary Methods and Figures

## Data accessibility

RNA-seq data are archived in NCBI GEO.
