# Supplementary material for: A new developmental mechanism for the separation of the mammalian middle ear ossicles from the jaw
Source: Proc Biol Sci. 2017 Feb 8;284(1848):20162416. doi: 10.1098/rspb.2016.2416 (PMC5310609; doi:10.1098/rspb.2016.2416)
Supplement: Supplementary Methods and Figures [file rspb20162416supp1.pdf]

Title: A New Developmental Mechanism for the Separation of the Mammalian Middle Ear Ossicles from the Jaw

Journal: *Proceedings of the Royal Society B: Biological Sciences*

Authors: Daniel J. Urban[1†], Neal Anthwal[3†], Zhe-Xi Luo[4], Jennifer A. Maier[1], Alexa Sadier[1], Abigail S. Tucker[3], and Karen E. Sears[1,2*]

Affiliations:

[1]School of Integrative Biology, 505 S Goodwin Avenue, University of Illinois, Urbana IL 61801 USA

[2] Carl Woese Institute for Genomic Biology, 1206 W Gregory Drive, University of Illinois, Urbana IL 61801 USA

[3]Department of Craniofacial Development and Stem Cell Biology, King's College London, London, UK

[3]Department of Organismal Biology and Anatomy, University of Chicago, Chicago IL 60637 USA

[†]These authors contributed equally to this work.

Correspondence to: Dr. Karen Sears, ksears2@illinois.edu

Article DOI: 10.1098/rspb.2016.2416

Supplementary Materials and Methods:

Immunofluorescence. In preparation for cryosectioning, specimens were rehydrated through a reverse MeOH series and rinsed in 1xPBS for 1.5 hours (with changes every 30 minutes). Afterward, they were sunk in 30% sucrose solution (in 1xPBS) and stored at 4 degrees C overnight. The following day, specimens were equilibrated in Optimal Cutting Temperature compound (OCT) at room temperature for 1-3 hours. Finally, they were transferred into a mold with fresh OCT and flash frozen in a mixture of dry ice and ethanol (EtOH). Frozen blocks were stored in -80 degrees C until cut. Specimens were sectioned in a Thermo Scientific Cryostat Microtom (Microm HM550) at a thickness of 10 microns. Sections were collected on superfrost glass slides and immediately stored on dry ice until transferred to a -20 degree freezer.

Sections from 16, 18, and 20 day opossums underwent immunofluorescence (IF) staining in order to highlight apoptosis, autophagy, and cellular proliferation (32, 33). IF protocol for cellular proliferation (using Phosphohistone H3 (Ser10) antibody from Cell Signaling Technology) and autophagy (using Anit-LC3B antibody (ab51520) from Abcam). Day 1 of protocol: blocking buffer (100mL 1xPBS + 1mL HIGS + 100ul Triton X-100), block for 10 min, gently dry, 100ul per slide phH3 1:100 concentration (100ul buffer + 1ul phH3) or LC3B at 1:1000 concentration (1mL buffer + 1ul LC3B), cover slip, humidified chamber in 4 degree C overnight (with excess buffer). Day 2 of protocol: 3x 20 washes with blocking buffer, 100ul secondary antibody per slide 1:250 concentration (300ul buffer + 1.2ul Goat anti-Rabbit (Alexa Fluor 488 conjugate from Invitrogen)), cover slip, humidified chamber at room temp for 1 hour (in the dark), 3x 20 min washes in buffer (keep in dark), gently dry, 2-3 drops Vectashield Mounting Medium with DAPI, store at 4 degrees C until imaging.

IF staining for cellular death was performed using the EMD Millipore ApopTag Fluorescein In Situ Apoptosis Detection Kit (S7110), an indirect TUNEL method (34, 35). Terminal deoxynucleotidyl transferase (TdT) modifies genomic DNA for detection of positive cells, and

an anti-digoxigenin antibody is conjugated to a fluorescent reporter. Protocol: fix slides in 1% PFA in 1xPBS (938ul of 16% PFA + 15mL PBS), 2x 5 min rinses in 1xPBS, post fix in pre-cooled 2:1 mix of EtOH:Acetic acid at -20 degrees C, 2x 5 min rinses in 1xPBS, gently dry excess liquid, apply 75ul of Equilibration Buffer per slide for 10 sec, remove excess liquid, apply 55ul of Working Strength TdT Enzyme per slide (77ul Reaction Buffer + 33ul TdT Enzyme), coverslip and place in humidified chamber for 1 hour at 37 degrees C, 10 min in Working Strength Stop/Wash Buffer (1mL stop/wash + 34mL ddH_2O), 3x 1 min washes in 1xPBS, apply 65ul Anti-Digoxigenin Conjugate per slide (62ul anti-Digo + 68ul Blocking Solution), coverslip and place in humidified chamber for 30 min at room temperature (keep in dark), 4x 2 min washes in 1xPBS, 2-3 drops of Vectashield Mounting Medium with DAPI (4',6-diamidino-2-phenylindole) per slide, coverslip and dry for several hours in the dark at room temperature, store at -20 degrees C. DAPI fluoresces when bound to DNA and is used as a nuclear counterstain.

All IF slides were imaged using a Leica Microsystems DMI4000 B automated inverted fluorescence microscope with a Hamamatsu ORCA-ER high-resolution digital camera, and using Image-Pro Plus 7.0 software. The Hamamatsu is a black and white camera so all images where taken three times, using blue, green, and red fluorescence. Afterwards, the separate color channels were merged into one, using FIJI/ImageJ software (NIH) (36).

RNA-sequencing. Additional samples were cryosectioned for the specific purpose of collecting tissue for RNA-Sequencing. N=3 specimens were collected for each stage (16, 18, and 20 day) and immediately snap-frozen, without fixation, then cryosectioned on Arcturus PEN membrane glass slides (Applied Biosystems), five slides per specimen. An Arcturus Veritas Microdissection Instrument was used for laser capture microdissection (LCM) of Meckel's cartilage and malleus (with minimal surrounding perichondrium) focusing on their connection area. An UV cutting laser was used to excise the tissue of interest. Afterward, an IR capture laser was fired through Arcturus Capsure HS LCM Caps, melting an attached transfer film, which would then bond with

the tissue. The cells attached to the caps were removed with an Arcturus PicoPure RNA Isolation Kit. An ExtracSure Extraction Device was placed on each cap and 10ul of Extraction Buffer (XB) was added to each. A 0.5mL microcentrifuge tube was placed on top, then covered with an incubation block preheated to 42 degrees C, and left to incubate for 30 min. Afterward, they were centrifuged for 2 min at 800x g, the extracted RNA was pooled with the other slides for each specimen, and the cell extract was stored at -80 degrees C. We also took slide scrapes for each set (used for baseline comparison) by placing RNA extraction buffer directly on the slides.

The remainder of the RNA isolation was completed following the PicoPure RNA Isolation Kit guidelines. RNA integrity was checked by an Agilent 2100 Bioanalyzer, revealing RIN values of 7.8 – 8.9. As the quantity of RNA in cartilage is commonly very low, we used the Clontech SMARTer Ultra Low Input RNA Kit for amplification. We began the Clontech amplification with 9ul of pooled RNA from each specimen. The first portion of this protocol, first-strand cDNA synthesis, was completed inside of a PCR clean hood workstation. The final portion, cDNA purification, was completed using Agencourt Ampure XP beads with a magnetic block for separation. All steps were conducted following standard Clontech Kit guidelines, with the final supernatant containing purified cDNA from the original tissue samples stored at -20 degrees C.

Resultant cDNA samples were run through the Bioanalyzer again, as well as a Qubit Fluorometer, to determine accurate concentrations. Next, we built libraries for sequencing using a Nextera XT DNA Sample Preparation Kit, along with a Nextera XT Index Kit and TruSeq Dual Index Sequencing Primers. All samples started with 5ul of input DNA at a concentration of 0.2ng/ul. The standard protocol was followed for the Nextera XT DNA Library Preparation Guide. DNA was tagged and fragmented, amplified via PCR, and cleaned up with AMPure XP beads to purify the library. Afterwards, the library was again validated on the Agilent 2100 Bioanalyzer, followed by library normalization and pooling for HiSeq sequencing. High-

throughput sequencing was conducted on an Illumina HiSeq 2500, at the W.M. Keck Center for Comparative and Functional Genomics at the University of Illinois (37).

Initial RNA-Seq analysis was conducted on the UIUC web-based Galaxy (38) platform (galaxy.illinois.edu), using the Tuxedo protocol. Sequence files were uploaded, along with an opossum reference genome (monDom5) (39) from ensemble.org. The basic sequence for analyses was as follows: Edit Sequences was used to trim ends, TopHat was used to align reads to the genome, Cufflinks was used to assemble the reads into transcripts, Cuffmerge was used to blend multiple samples from the same stage, and Cuffdiff was used to report genes and transcripts that are differentially expressed between samples (40). We also used the Database for Annotation, Visualization and Integrative Discovery (DAVID) Bioinformatics Resource (david.ncifcrf.gov) (41) to identify Gene Ontology (GO) terms describing gene functions for our list of differentially expressed genes and their functionally related gene groups.

In situ hybridization - Select genes identified from RNA-Seq were confirmed via fluorescence *in situ* hybrization (FISH) (42), using cryosectioned slides from 16, 18, and 20 day specimens. FISH probes for *TGFbr2* and *WISP1* were designed and manufactured by Molecular Instruments. Each set of probes consists of 5x 20pmole probes with 2x fluorophore labeled hairpins (Alexa488). Protocol was as follows: Day 1- thaw slides, draw hydrophobic circle around tissues with a PAP pen, rinse with DEPC water, 0.2M acid hydrolysis for 15 min (833ul 12M HCL in 50mL DEPC water), wash 2x 5 min in DEPC PBS, wash 1x 6 min in 1ug/mL Proteinase K (1ul in 10mL DEPC PBS), wash 1x 10 min in DPEC PBS, fix with 4% DEPC PFA for 5 min, wash 2x 5 min in DEPC PBS, acetylation with 0.25% acetic acid for 10 min (25ul glacial acetic acid in 10mL DEPC water), wash 2x 5 min in DEPC PBS, pre-hybridize in probe hybridization buffer for 15 min at 45 degrees C, prepare the probe solution (thaw 5 probes on ice, mix 1ul of each probe in 500ul of probe hybridization buffer, warm mixture to 45°C), remove pre-hybridization solution and add probe solution (~150-200ul per slide), cover slip with

parafilm, incubate in humidified chamber overnight (>12 hours) at 45 degrees C. Day 2- remove excess probe with wash buffer (perform washes at 45 degrees C), ~300-500ul of wash buffer per slide, 2x 5 min washes, 2x 30 min washes, 1x 5 min wash, pre-amplify samples in 500ul of amplification buffer for 30 min at room temperature, "snap cool" 2x hairpins (10ul each hairpin in individual PCR tubes, 95 degrees C for 90 seconds in thermal cycler, cool to room temperature in dark drawer for 30 min), mix both hairpins together with 500ul of amplification buffer, remove pre-amplification solution and add hairpin solution (~150-200ul per slide), cover slip with parafilm, incubate in dark overnight (>12 hours) at room temperature. Day 3- remove excess hairpins with 5x SSCT (5x SSC with Tween, 10ul Tween per 10mL of 5x SSC) washes at room temperature, 2x 5 min washes, 2x 30 min washes, 1x 5 min wash, 2-3 drops of Vectashield Mounting Medium with DAPI per slide, glass coverslip, store at 4 degrees C until imaging.

Functional Assays - After identifying *TGFbr2* as having a potential role in Meckel's cartilage separation, we knocked down *TGFb* signaling to investigate the impact on phenotype. A TGFb1,2,3 antibody (MAB1835) from R&D Systems was used to neutralize the biological activity of *TGFb* signaling (43). The antibody was reconstituted at 0.5mg/mL in sterile PBS, and 38ul intraperitoneal (IP) injections (based upon estimated 1.9g weight of pups at 20 days) were administered to neonatal opossum for six consecutive days beginning on postnatal day 16. Control pups were injected with an equal dosage of 1xPBS. Mothers were anesthetized with isoflurane during injections. This precluded the necessity of physically removing the pups from the mothers, as the pups are continually attached to the mother's nipples at this developmental stage. We utilized a precision vaporizer isoflurane anesthesia machine with the oxygen flowmeter set to 1 litter per minute (LPM) and the vaporizer dial set to 2½. Mothers were placed in an induction chamber until they lost consciousness, at which point they were switched to a nose cone for the remaining duration. Injections took less than five minutes and the mothers fully recovered within a few minutes after returning to their cages. Pups were euthanized on postnatal day 22. MC morphology was visualized using micro-CT scanning (as described above) and

clearing and staining for at least 3 control and 3 treatment pups. For clearing and staining, following 4% PFA fixation and dehydration into ethanol, P22 samples were placed in acetone, then stained with 0.3% Alcian Blue 8GX (A5268 Sigma) and 0.1% Alizarin Red S (A5533) at 37° C for 5 days. Samples were then cleared in a 1% KOH solution (changed daily) for ~2 weeks. Once cleared, specimens were imaged using a Leica M205 C stereo microscope with a Leica DFC425 digital camera, utilizing the Leica Application Suite (LAS) version 3.8. At least 3 treatment and 3 control pups were also sectioned and IF used to test for apoptotic cells (as described above) and anti-p-Smad2 (44) (at a working concentration of 1:100; otherwise as described above for the Phosphohistone H3 antibody) (Cell Signaling Technology).

Supplementary Figures:

Fig S1. A - Micro-CT scan image of postnatal day (P) 20 opossum skull with elements relating to the middle ear colored (MC = light green, ectotympanic = purple, goniale = dark green, malleus = light blue, incus = dark blue, and stapes = red). At P20, Meckel's cartilage (MC, light green) rests within the gonial trough (dark green) and is just beginning to detach from the malleus (light blue). B & C – Isolated middle ear structures at P20 in lateral (B) and dorsal (C) view. D – K - Micro-CT scan images of developing opossum skulls at five day increments, beginning on the day of birth through P35, with the middle ear elements colored as in (A). Black arrows indicate separation of MC from the malleus, which first occurs at P20.

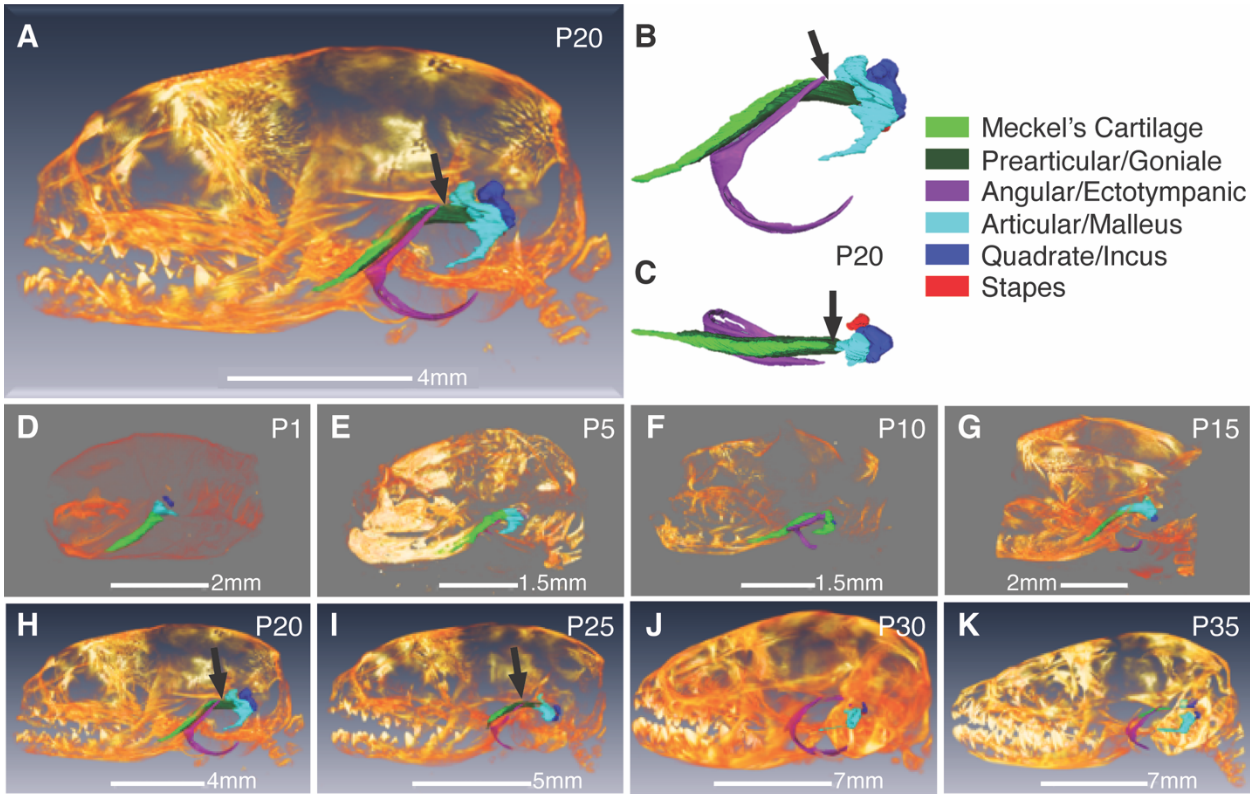

Fig S2. Laser tissue capture microdissection was used to extract tissues for RNA-seq. Shown are a representative section before (A) and immediately after (B) laser tissue capture microdissection, and the resultant tissue section that was used for RNA-seq (C).

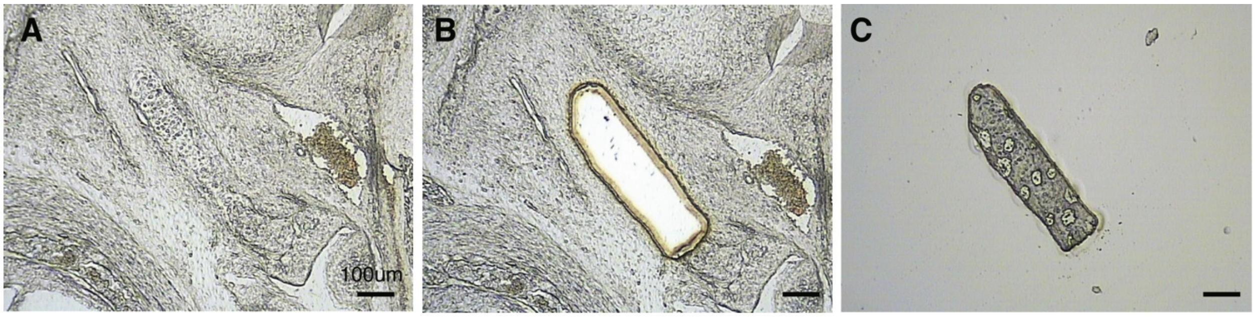

Fig S3. Cryosectioned and IF stained slides of the middle ear region. A-D - pSMAD IF stained (green) slides of the comparable ear regions of *TGF-β* neutralizing-antibody treated (A, B, different sections showing the same assay) and control (C, D, different sections showing the same assay) opossums at postnatal day (P) 22, counterstained with the nuclear stain DAPI (blue). pSMAD staining, in this case, is used as a read-out for TGFB signaling. All scale bars = 100um. pSMAD staining is qualitatively more abundant in control (C, D) than *TGF-β* neutralizing-antibody treated (A, B) specimens. (E) The number of pSMAD positive cells was also counted for 11 control and 11 *TGF-β* neutralizing-antibody treated images (taken at the same magnification). Results indicate that there are significantly more pSMAD positive cells in control than *TGF-β* neutralizing-antibody treated images ($P < 0.0001$*). F-G – Positive controls for IF for proliferation (F) and autophagy (G) in opossum middle ear tissues. Representative positive cells are indicated with white arrows. Alt sxn = alternative section.

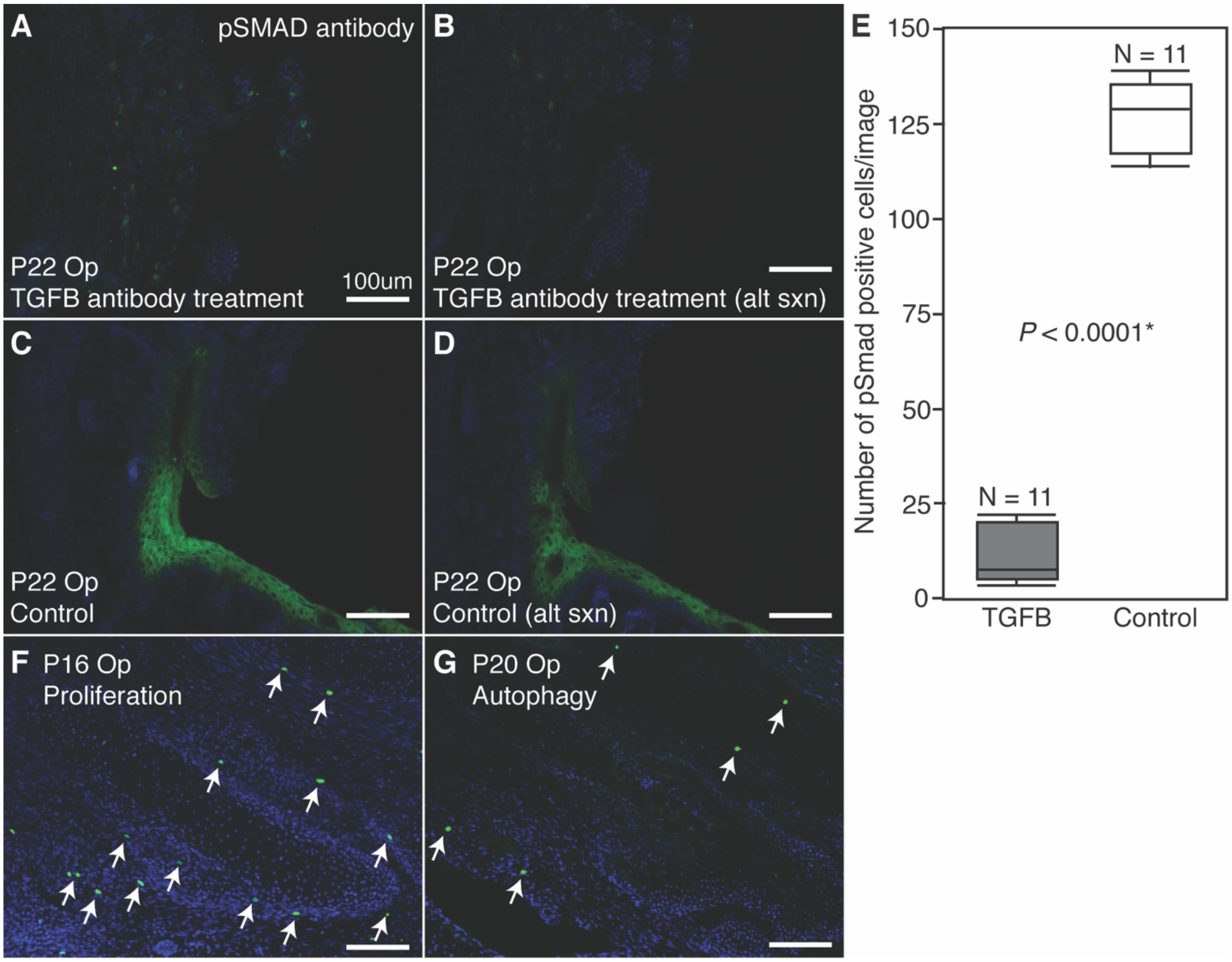
A
pSMAD antibody
P22 Op
TGFB antibody treatment
100um
B
P22 Op
TGFB antibody treatment (alt sxn)
C
P22 Op
Control
D
P22 Op
Control (alt sxn)
E
150
125
100
75
50
25
0
Number of pSmad positive cells/image
N = 11
N = 11
P < 0.0001*
TGFB
Control
F
P16 Op
Proliferation
G
P20 Op
Autophagy

Fig. S4. Micro-CT reconstructions in side profile (A-B) and dorsally in conjunction with 2D orthoslice without (C-D) and with (E-F) middle ear elements highlighted in P22 opossum pups. Cleared and stained middle ear structures in side profile (G-H) and dorsal view (I-J). TNA treated pups are shown in A, C, E, G, and I, and control pups in B, D, F, H, and J. A-B – Micro-CT reconstructions with ossified structures of skull more defined (i.e. less opaque) show no pleiotropic deformations caused by TNA treatment. C-F – Dorsal views with 2D slice illustrates how the goniale cradles the MC, and is clearly differentiated from the MC as the goniale is ossified at this point. G-J – Cleared and stained samples show that the posterior end of MC (indicated by black arrows) is detached and partially degraded in control but not TNA specimens. Additionally, the images show no apparent disruption of goniale phenotype.

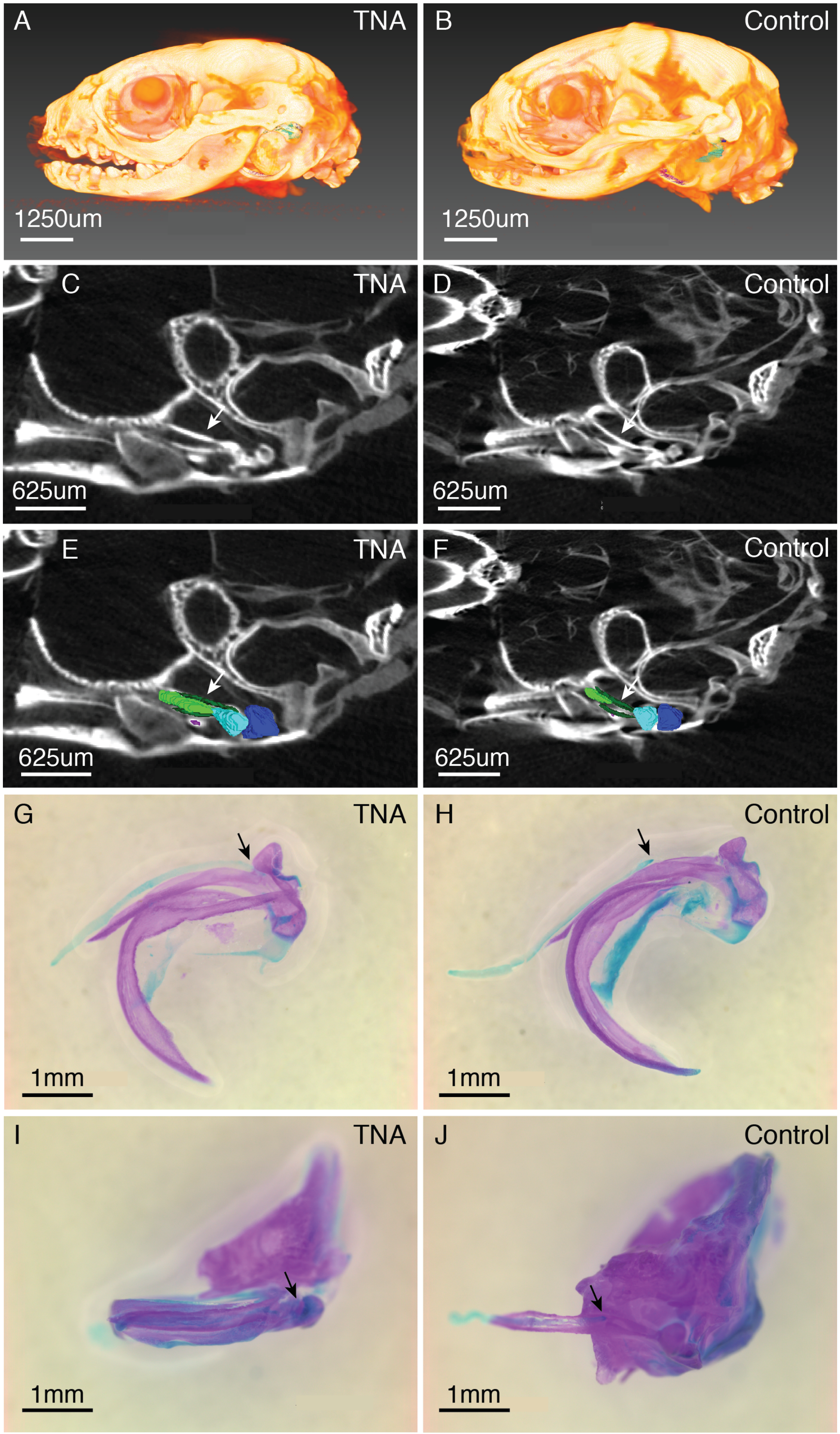
A
TNA
1250um
B
Control
1250um
C
TNA
625um
D
Control
625um
E
TNA
625um
F
Control
625um
G
TNA
1mm
H
Control
1mm
I
TNA
1mm
J
Control
1mm

Fig S5. Alternative hypothesis of homoplastic evolution of Mammalian Middle Ears under *TGF-β* signaling. Although the Meckel's sulcus is present in all zatherian stem taxa to crown therians, and in some stem eutherians and metatherians, the ossified Meckel's cartilage itself has not been preserved in these fossils. If treating this as an absence of data, or as presence/absence indeterminate (= "?"), then it is feasible to hypothesize that the theriiform clade (multituberculates + crown therians) had possessed the DMME condition with MC breakdown ancestrally, as documented in multiple multituberculates. If so, an additional evo-devo scenario would also be possible - the presence of ossified Meckel's cartilage in *Maotherium* could be a hypothetical reversal on evolutionary tree, mechanistically possible by a down regulation of gene(s) involved in *TGF-β* signaling (such as *TGF-βR2)* as in the antibody treatment of *TGF-βR2.* It could not be excluded that separate gains of MC breakdown (in monotremes and in theriiforms), and its reversal (only in Maotherium) all occurred in the early history of mammals, given the homoplastic nature of *TGF-β* signaling and its multiple down-cascade developmental processes. Another scenario would be that the Meckel's cartilage was lost in the last common ancestor of marsupials and placentals (the Theria node), following the interpretation that *Peramus* and other stem zatherians retained a MC in the Meckel's sulcus (*53-58*). However, because the Meckel's sulci in stem zatherians are identical to those of basal eutherians (*53-55*) and the metatherian *Kokopelia* (*56*), it is not parsimonious to interpret them differently. Thus we consider the latter scenario to be less likely. All evo-devo hypotheses (Fig. 3 vs. Fig. S5) are contingent on the interpretation of the presence/absence of the MC and its osteological correlates on the dentary in stem eutherians and metatherians, and their immediate zatherian outgroups. But current evidence is more favorable for the scenario presented in Fig 3 than in Fig S5. Our preferred hypothesis is that parallel up regulations of *TGF-β* signaling occurred in multiple early mammal lineages, facilitating the independent acquisitions (Fig 3). However, if the scenario in Fig S5 is correct, then this would suggest that significant developmental systems drift has occurred in modern marsupial and placental mammals. (drawings of *Kokopelia* and *Peramus* courtesy of R. L. Cifelli and B. M. Davis.)

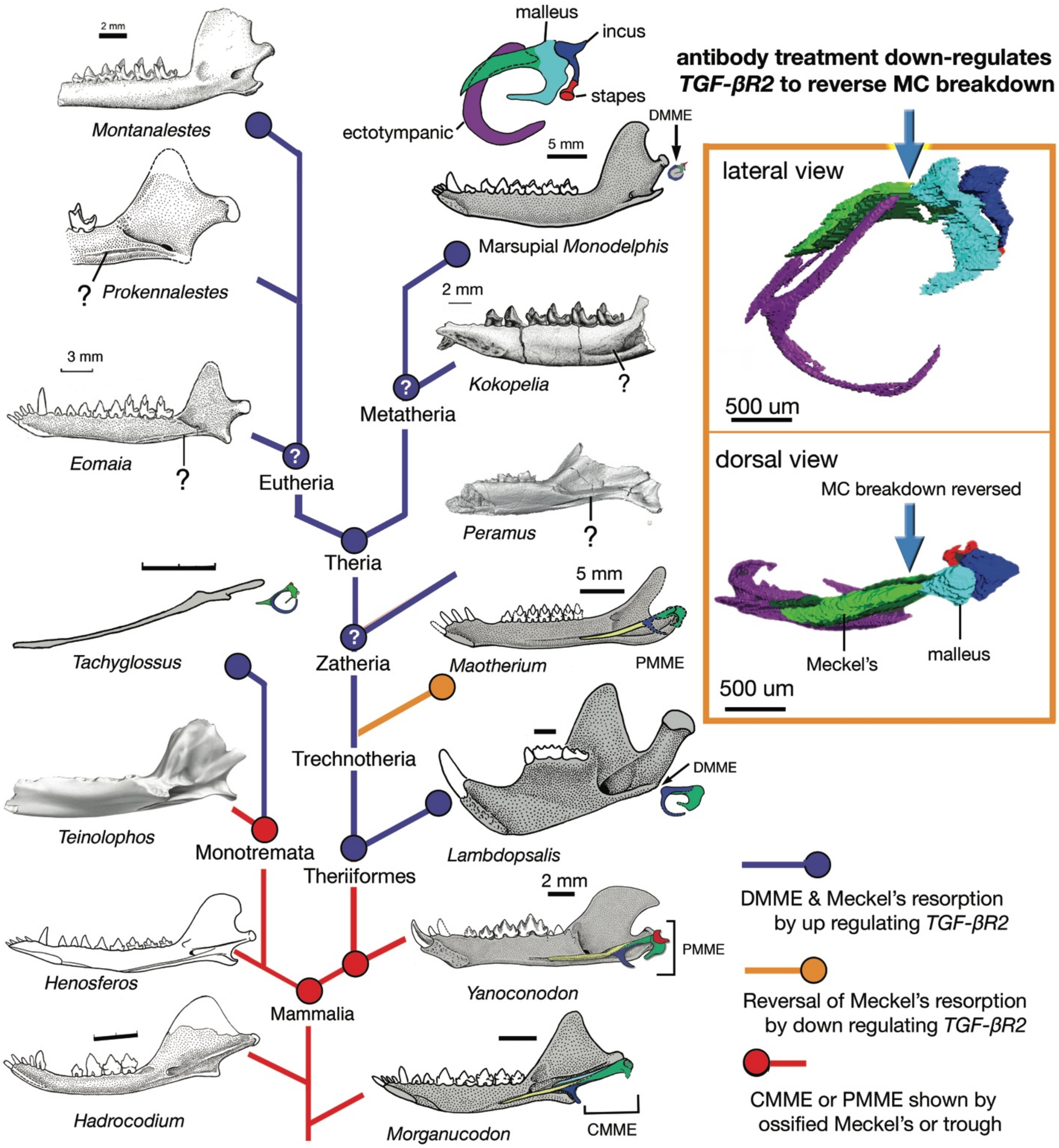

2 mm
Montanalestes
? Prokennalestes
3 mm
Eomaia
?
Eutheria
malleus
incus
stapes
ectotympanic
DMME
5 mm
Marsupial Monodelphis
2 mm
Kokopelia
?
Metatheria
Peramus
?
Theria
antibody treatment down-regulates TGF-βR2 to reverse MC breakdown
lateral view
500 um
dorsal view
MC breakdown reversed
Meckel's
malleus
500 um
Tachyglossus
Zatheria
5 mm
Maotherium
PMME
Trechnotheria
DMME
Teinolophos
Monotremata
Theriiformes
Lambdopsalis
2 mm
PMME
Henosferos
Mammalia
Yanoconodon
Hadrocodium
Morganucodon
CMME
DMME & Meckel's resorption by up regulating TGF-βR2
Reversal of Meckel's resorption by down regulating TGF-βR2
CMME or PMME shown by ossified Meckel's or trough

Tables:

Table S1. Genes that are differentially expressed in the opossum Meckel's cartilage and its perichondrium by a log-fold change of ≥2 at and before P20 (Sample1 = P16 or P18, and Sample2 = P20), as identified by RNA-seq.

Gene Name	Sample1	Value1	Sample2	Value2	log2(fold_change)	P_value
CRYGN	P16	0.8324	P20	75.8965	6.5107	0.0003
SLC30A1	P16	0.3747	P20	20.4888	5.7728	0.0001
CRYGN	P18	1.6332	P20	75.8965	5.5383	0.0002
MOB3A	P16	0.2553	P20	9.2157	5.1739	0.0003
GPNMB	P16	5.4177	P20	193.4330	5.1580	0.0001
F10	P16	0.3024	P20	9.6712	4.9991	0.0002
HEYL	P16	0.2930	P20	7.5006	4.6780	0.0003
CLIC2	P16	0.3666	P20	8.8372	4.5913	0.0005
SLC13A5	P16	0.9642	P20	23.2089	4.5892	0.0001
LAT	P16	1.6472	P20	39.5272	4.5848	0.0004
PHLDA3	P16	3.1026	P20	71.7884	4.5322	0.0001
MRGPRF	P16	0.3743	P20	8.5915	4.5205	0.0004
ABCB8	P16	1.2166	P20	26.8737	4.4652	0.0001
ABCD4	P16	0.2849	P20	6.2188	4.4483	0.0006
TOR1B	P16	0.3877	P20	8.2769	4.4159	0.0004
ANAPC2	P16	0.4689	P20	9.8237	4.3890	0.0001
CHN2	P16	0.6339	P20	13.2001	4.3801	0.0001
PEX19	P16	1.5901	P20	33.0957	4.3794	0.0004
CAP1	P16	4.6801	P20	97.1644	4.3758	0.0003
SESN1	P16	0.9021	P20	17.6288	4.2884	0.0001

RND3	P16	0.9627	P20	18.3046	4.2490	0.0003
PEX5	P16	0.4076	P20	7.3453	4.1714	0.0001
IRAK2	P16	0.5860	P20	10.3899	4.1483	0.0001
FGF1	P16	0.9807	P20	17.3449	4.1445	0.0002
IL13RA1	P16	0.5382	P20	9.3901	4.1249	0.0002
COL10A1	P16	2.3641	P20	40.9610	4.1149	0.0004
NHLRC3	P16	0.9749	P20	16.8160	4.1084	0.0003
RELL1	P16	0.7694	P20	13.1456	4.0948	0.0003
PLA1A	P16	0.2976	P20	5.0599	4.0878	0.0006
CA7	P16	4.5772	P20	76.6977	4.0667	0.0001
LYRM2	P16	2.6861	P20	44.9668	4.0653	0.0004
LDHB	P16	7.4146	P20	123.9810	4.0636	0.0005
CYB5R1	P16	2.4520	P20	40.7501	4.0548	0.0004
ATP6V1H	P16	1.9625	P20	32.1239	4.0329	0.0002
CYP1B1	P16	2.3490	P20	38.1663	4.0222	0.0001
PTPRJ	P16	0.7220	P20	11.0466	3.9355	0.0002
CAPN1	P16	0.6118	P20	9.3096	3.9275	0.0002
TBC1D8B	P16	0.2400	P20	3.6126	3.9122	0.0005
WFDC1	P16	5.9516	P20	88.6521	3.8968	0.0002
TMEM176A	P16	2.3058	P20	33.8702	3.8767	0.0004
SMPD1	P16	7.9985	P20	116.4700	3.8641	0.0006
FBXL5	P16	0.7049	P20	10.2397	3.8605	0.0002
SRPX2	P16	1.9418	P20	27.3680	3.8170	0.0001
RNF149	P16	1.5006	P20	21.0959	3.8133	0.0003
UBALD1	P18	0.2886	P20	3.9707	3.7823	0.0006

SH3GLB1	P16	4.5504	P20	61.9177	3.7663	0.0004
PSTPIP1	P16	3.8211	P20	51.7928	3.7607	0.0005
TEX264	P16	2.5089	P20	33.9832	3.7597	0.0006
ATP6V1G1	P16	14.2728	P20	191.9410	3.7493	0.0001
LDHA	P16	4.8011	P20	62.3381	3.6987	0.0001
NPEPL1	P16	1.7956	P20	23.2428	3.6943	0.0002
GUSB	P16	18.2832	P20	236.2410	3.6917	0.0002
TGFBR2	P16	1.7625	P20	22.7159	3.6880	0.0001
CSTB	P16	28.9600	P20	356.9720	3.6237	0.0001
NELFE	P16	4.4724	P20	55.0475	3.6216	0.0004
SLC15A4	P16	7.0713	P20	86.0409	3.6050	0.0002
GNPTAB	P16	6.8039	P20	82.6563	3.6027	0.0001
ATP6V1A	P16	7.1738	P20	86.1035	3.5853	0.0001
RPS6KB2	P16	11.1784	P20	132.9680	3.5723	0.0004
PSMD11	P16	3.6688	P20	43.4043	3.5645	0.0001
EMC3	P16	3.0389	P20	35.8801	3.5616	0.0004
AAGAB	P16	1.6075	P20	18.6800	3.5386	0.0003
EIF2A	P16	1.4181	P20	16.4793	3.5386	0.0005
ACAD9	P16	1.5728	P20	18.0065	3.5171	0.0006
ADAM15	P16	2.1646	P20	24.4993	3.5006	0.0001
PFKL	P16	3.1802	P20	35.5393	3.4822	0.0001
ANKH	P16	5.9964	P20	66.6633	3.4747	0.0004
LCP1	P16	3.2972	P20	36.1764	3.4557	0.0001
CERCAM	P16	5.1337	P20	55.4056	3.4320	0.0001
ALPL	P16	27.7116	P20	296.4290	3.4191	0.0001

SPP1	P16	338.7830	P20	3610.3400	3.4137	0.0004
NDUFS1	P16	2.1621	P20	22.8393	3.4010	0.0001
FOS	P16	2.5663	P20	27.0343	3.3970	0.0001
FBXW5	P16	3.0730	P20	32.3598	3.3965	0.0001
ZNF212	P18	0.9259	P20	9.7384	3.3947	0.0003
ISCA1	P16	1.8158	P20	19.0385	3.3903	0.0002
PHC2	P16	1.9818	P20	20.6799	3.3833	0.0001
DOHH	P16	4.1690	P20	43.3923	3.3797	0.0001
CDKN1A	P16	2.6423	P20	27.4103	3.3749	0.0002
PAMR1	P16	4.8940	P20	50.7366	3.3740	0.0006
ALDH1L2	P16	2.1907	P20	22.7000	3.3732	0.0001
LGMN	P16	5.0190	P20	51.9234	3.3709	0.0006
EXOSC10	P16	1.3799	P20	14.1384	3.3570	0.0002
THOC3	P16	1.7299	P20	17.6879	3.3540	0.0001
RAB38	P16	5.5933	P20	56.3397	3.3324	0.0001
TTLL9	P18	4.0606	P20	40.8482	3.3305	0.0001
NQO1	P16	7.9475	P20	79.9176	3.3299	0.0001
TGFBI	P16	8.1163	P20	81.6119	3.3299	0.0001
CD200	P16	7.7808	P20	78.2029	3.3292	0.0003
C1orf198	P16	1.3587	P20	13.6550	3.3292	0.0001
ZNF212	P16	0.9826	P20	9.7384	3.3090	0.0003
C16orf72	P16	2.4425	P20	24.1595	3.3062	0.0001
AGFG1	P16	0.8293	P20	8.1228	3.2920	0.0002
CCNG1	P16	5.6563	P20	55.0936	3.2840	0.0001
BLVRB	P16	6.3890	P20	62.2087	3.2835	0.0004

SMIM14	P16	2.4568	P20	23.8276	3.2778	0.0004
SEC23IP	P16	0.8076	P20	7.8010	3.2720	0.0001
EI24	P16	6.7958	P20	65.5888	3.2707	0.0001
SRM	P16	8.8766	P20	85.5215	3.2682	0.0001
CMAS	P16	3.8087	P20	36.4443	3.2583	0.0001
MAMDC2	P16	1.1091	P20	10.5743	3.2531	0.0001
DUSP7	P16	2.0387	P20	19.3555	3.2470	0.0002
IL21R	P18	1.2314	P20	11.6860	3.2464	0.0004
HERPUD1	P16	4.2197	P20	39.8036	3.2377	0.0001
MME	P16	9.7353	P20	91.7013	3.2356	0.0003
TMEM127	P16	4.2297	P20	39.7176	3.2312	0.0004
ELP6	P16	1.9598	P20	18.3946	3.2305	0.0003
NDFIP1	P16	11.4032	P20	105.0680	3.2038	0.0002
BGLAP	P16	339.9940	P20	3088.9700	3.1835	0.0001
UBE2O	P16	1.1107	P20	9.9429	3.1622	0.0004
LEMD2	P16	1.7026	P20	15.1901	3.1573	0.0003
EEF2	P16	2.9179	P20	25.7080	3.1392	0.0006
COPG1	P16	4.5783	P20	40.0315	3.1282	0.0002
RAP1B	P16	5.2698	P20	45.8499	3.1211	0.0004
ATP6V0E1	P16	16.7800	P20	144.0400	3.1017	0.0002
ATP5A1	P16	13.9893	P20	119.9680	3.1003	0.0001
STAT3	P16	3.1351	P20	26.8130	3.0964	0.0001
COPS4	P16	3.5697	P20	30.4541	3.0928	0.0005
ACAA1	P16	25.4232	P20	216.4430	3.0898	0.0002
SHROOM4	P16	0.7053	P20	5.9220	3.0698	0.0001

RPN2	P16	12.5821	P20	105.5130	3.0680	0.0003
TWISTNB	P16	5.0284	P20	42.1052	3.0658	0.0002
EDEM2	P16	3.0920	P20	25.8693	3.0646	0.0002
LSM6	P16	1.6086	P20	13.4273	3.0613	0.0004
RPS27L	P16	4.7378	P20	39.5277	3.0606	0.0003
ADAM15	P18	2.9377	P20	24.4993	3.0600	0.0002
DNAJB11	P16	13.6788	P20	114.0590	3.0598	0.0002
CRAT	P18	2.9422	P20	24.5235	3.0592	0.0005
U6	P16	1.5852	P20	13.0980	3.0466	0.0002
BHLHE40	P16	1.4196	P20	11.6905	3.0418	0.0002
MRPS34	P16	3.7075	P20	30.4121	3.0361	0.0003
WISP1	P16	8.6652	P20	70.6432	3.0273	0.0001
TRIM35	P16	1.1764	P20	9.5519	3.0214	0.0002
LGALS3	P16	7.1541	P20	57.7651	3.0134	0.0001
RTN4	P16	10.3529	P20	83.5046	3.0118	0.0004
DERL1	P16	2.7446	P20	22.1139	3.0103	0.0001
NDUFA8	P16	8.9907	P20	72.4168	3.0098	0.0002
YIPF3	P16	18.6898	P20	150.4320	3.0088	0.0002
PDZD8	P16	1.2030	P20	9.6810	3.0086	0.0002
ARL6IP5	P16	18.6137	P20	149.4410	3.0051	0.0001
TOR1A	P16	2.4346	P20	19.5142	3.0028	0.0002
SLC25A3	P16	19.9596	P20	159.5180	2.9986	0.0001
GGT5	P16	9.2224	P20	73.4474	2.9935	0.0003
CYBA	P16	43.3230	P20	343.1310	2.9856	0.0003
MRPL17	P16	2.2576	P20	17.8413	2.9824	0.0003

IPO9	P18	1.8207	P20	14.3420	2.9777	0.0002
CAT	P16	3.2957	P20	25.9307	2.9760	0.0004
TIMP2	P16	3.8181	P20	29.8808	2.9683	0.0003
COL5A2	P16	59.3856	P20	464.7180	2.9682	0.0003
ACTR10	P16	1.2681	P20	9.8580	2.9586	0.0004
THOC3	P18	2.2755	P20	17.6879	2.9585	0.0002
LTBR	P16	6.3426	P20	49.1095	2.9529	0.0003
PRSS23	P16	4.4049	P20	34.0243	2.9494	0.0001
AAR2	P16	0.9889	P20	7.6348	2.9487	0.0004
CDH15	P16	1.8866	P20	14.5234	2.9445	0.0003
MXD4	P16	5.2881	P20	40.1154	2.9233	0.0002
PDLIM7	P16	13.1429	P20	99.4072	2.9191	0.0002
TIMM50	P16	2.1672	P20	16.3617	2.9164	0.0003
SPNS1	P16	4.6489	P20	35.0264	2.9135	0.0004
LRPAP1	P16	21.9156	P20	164.5920	2.9089	0.0001
GAS1	P16	1.4824	P20	11.0917	2.9035	0.0003
PDIA5	P16	3.3190	P20	24.6629	2.8935	0.0001
HIF1A	P16	3.8875	P20	28.7906	2.8887	0.0002
CORO1B	P16	2.6870	P20	19.8885	2.8879	0.0005
HM13	P16	18.4673	P20	136.5720	2.8866	0.0005
TMED7TICAM2	P16	3.3149	P20	24.3223	2.8753	0.0001
DYNLT3	P18	2.1378	P20	15.6742	2.8742	0.0003
LDLRAP1	q1	1.8955	P20	13.7809	2.8620	0.0002
MRPL44	q1	1.2940	P20	9.3757	2.8571	0.0005
RAP2A	q1	1.0080	P20	7.2773	2.8519	0.0006

EIF4H	q1	5.3779	P20	38.6516	2.8454	0.0004
CD44	q1	2.6598	P20	19.0495	2.8404	0.0001
SLC38A2	q1	4.1330	P20	29.5496	2.8379	0.0001
TP53INP1	q1	1.4336	P20	10.2464	2.8374	0.0005
ANXA2	q1	114.7870	P20	815.9270	2.8295	0.0004
SLC6A6	q1	4.1070	P20	29.1402	2.8268	0.0006
SMURF2	q1	0.6967	P20	4.9223	2.8208	0.0003
BTG1	q1	30.3017	P20	213.9780	2.8200	0.0001
ACTR1B	q1	2.6992	P20	19.0422	2.8186	0.0003
SHROOM4	q2	0.8410	P20	5.9220	2.8159	0.0003
SLC30A1	q2	2.9107	P20	20.4888	2.8154	0.0001
KCTD17	P16	2.3516	P20	16.3364	2.7964	0.0003
PEX12	P16	3.3597	P20	23.3272	2.7956	0.0004
TSC22D1	P16	13.0848	P20	90.0301	2.7825	0.0003
CNOT10	P16	1.6229	P20	11.1560	2.7812	0.0004
KDELR2	P16	7.8427	P20	53.6487	2.7741	0.0001
ATP2B1	P16	4.0235	P20	27.1793	2.7560	0.0006
SGSM3	P18	4.4041	P20	29.6479	2.7510	0.0001
DCAF7	P16	3.6234	P20	24.3811	2.7504	0.0004
GANAB	P16	8.0740	P20	54.2670	2.7487	0.0006
SERP1	P16	9.7854	P20	65.7051	2.7473	0.0002
PRKAB1	P16	1.6513	P20	11.0815	2.7465	0.0002
SNAI2	P16	15.4730	P20	103.7610	2.7454	0.0005
SNX2	P16	9.9401	P20	66.5531	2.7432	0.0001
CAPZA1	P16	10.9147	P20	73.0238	2.7421	0.0003

CYP1B1	P18	5.7072	P20	38.1663	2.7415	0.0003
UBE2B	P16	25.7263	P20	171.7000	2.7386	0.0005
PCMT1	P16	6.5489	P20	43.6257	2.7359	0.0001
GPC1	P16	2.9899	P20	19.7578	2.7243	0.0005
ARCN1	P16	5.8986	P20	38.8380	2.7190	0.0001
CRYZ	P16	13.2038	P20	86.5681	2.7129	0.0001
FKBP14	P16	23.6648	P20	154.7210	2.7089	0.0001
RNF149	P18	3.2361	P20	21.0959	2.7046	0.0003
CKAP4	P16	17.4912	P20	113.6620	2.7001	0.0001
PHGDH	P16	5.1652	P20	33.5483	2.6993	0.0005
TFE3	P16	4.6872	P20	30.3308	2.6940	0.0006
LAMC1	P16	2.4734	P20	15.9447	2.6885	0.0003
ARF4	P16	14.2977	P20	92.0415	2.6865	0.0002
KIAA2013	P16	3.1185	P20	19.8441	2.6698	0.0005
HPGD	P16	2.7963	P20	17.7919	2.6697	0.0004
MAT2A	P16	5.2932	P20	33.6077	2.6666	0.0006
GPNMB	P16	5.4177	P20	34.2538	2.6605	0.0003
TPBG	P16	4.6269	P20	29.1919	2.6575	0.0002
PIGT	P16	22.0115	P20	138.5010	2.6536	0.0003
RBM8A	P16	5.6275	P20	35.0975	2.6408	0.0001
MDFI	P16	7.6713	P20	47.7377	2.6376	0.0004
OSTC	P16	24.7118	P20	151.6430	2.6174	0.0001
PNRC1	P16	9.4137	P20	56.8202	2.5936	0.0002
PRKCDBP	P16	6.9837	P20	42.0017	2.5884	0.0002
JAG1	P16	0.6121	P20	3.5495	2.5358	0.0004

EPRS	P16	6.8668	P20	39.6481	2.5295	0.0002
EFCAB14	P16	5.0970	P20	29.4241	2.5293	0.0005
GPX7	P16	5.9311	P20	34.2052	2.5278	0.0002
EIF3D	P16	15.4771	P20	88.6152	2.5174	0.0006
CALU	P16	22.6371	P20	128.6070	2.5062	0.0006
LMAN1	P16	6.2334	P20	33.6614	2.4330	0.0002
LUM	P16	53.1506	P20	283.6810	2.4161	0.0003
CRTAP	P16	10.9128	P20	58.1254	2.4132	0.0001
ANXA1	P16	8.9166	P20	47.4461	2.4117	0.0003
WDR1	P16	6.6354	P20	34.0139	2.3579	0.0003
EIF3E	P16	11.2570	P20	57.5368	2.3537	0.0003
DYNC1H1	P16	1.4542	P20	7.2336	2.3145	0.0005
SLK	P16	2.6246	P20	12.9263	2.3001	0.0002
SFRP2	P16	11.4798	P20	54.9579	2.2592	0.0006
COL5A1	P16	7.8880	P20	37.6069	2.2533	0.0006
PSMA6	P16	11.2671	P20	53.5942	2.2500	0.0004
PSMC4	P16	8.4349	P20	39.4978	2.2273	0.0003
RPS27A	P16	270.8450	P20	1219.8700	2.1712	0.0001

References:

32. Cooper KL, Sears KE, Uygur A, Maier JA, Baczkowski KS, *et al.* (2014) Patterning and post-patterning modes of evolutionary digit loss in mammals. *Nature* 511(7507):41-45.

33. Dowling A, Doroba C, Maier JA, Cohen L, Vandeberg J, *et al.* (2016) Cellular and molecular drivers of differential organ growth: insights from the limbs of *Monodelphis domestica*. *Dev Genes Evol*:1-9.

34. Molineaux AC, Maier JA, Schecker T, & Sears KE (2015) Exogenous retinoic acid induces digit reduction in opossums (*Monodelphis domestica*) by disrupting cell death and proliferation, and apical ectodermal ridge and zone of polarizing activity function. *Birth Defects Res A Clin Mol Teratol* 103(3):225-234.

35. Hübler M, Niswander L, Peters J, & Sears KE (2010) The developmental reduction of the marsupial coracoid: A case study in *Monodelphis domestica*. *J Morphol* 271(7):769-776.

36. Schindelin J, Arganda-Carreras I, Frise E, Kaynig V, Mongair M, *et al.* (2012) Fiji: an open-source platform for biological-image analysis. *Nature Methods* 9(7):676-682.

37. Sears KE, Maier JA, Rivas-Astroza M, Poe R, Zhong S, *et al.* (2015) The relationship between gene network structure and expression variation among individuals and species. *PLoS Genet* 11(8):e1005398.

38. Afgan E, Baker D, van den Beek M, Blankenberg D, Bouvier D, *et al.* (2016) The Galaxy platform for accessible, reproducible and collaborative biomedical analyses: 2016 update. *Nucleic Acids Res*:gkw343.

39. Mikkelsen TS, Wakefield MJ, Aken B, Amemiya CT, Chang JL, *et al.* (2007) Genome of the marsupial *Monodelphis domestica* reveals innovation in non-coding sequences. *Nature* 447(7141):167-177.

40. Trapnell C, Roberts A, Goff L, Pertea G, Kim D, *et al.* (2012) Differential gene and transcript expression analysis of RNA-seq experiments with TopHat and Cufflinks. *Nature Protoc* 7(3):562-578.

41. Huang DW, Sherman BT, & Lempicki RA (2009) Systematic and integrative analysis of large gene lists using DAVID bioinformatics resources. *Nature Protoc* 4(1):44-57.

42. Maier JA, Lo YT, & Harfe BD (2013) *Foxa1* and *Foxa2* are required for formation of the intervertebral discs. *PloS one* 8(1):e55528.

43. Dasch JR, Pace DR, Waegell W, Inenaga D, & Ellingsworth L (1989) Monoclonal antibodies recognizing transforming growth factor-beta. Bioactivity neutralization and transforming growth factor beta 2 affinity purification. *J Immunol* 142(5):1536-1541.

44. Anthwal N, Chai Y, & Tucker AS (2008) The role of transforming growth factor-β signalling in the patterning of the proximal processes of the murine dentary. *Dev Dyn* 237(6):1604-1613.
